# Coronary Stenting in High Bleeding Risk Patients With Small Coronary Arteries Followed by One-Month Dual Antiplatelet Therapy: Onyx ONE Clear

**DOI:** 10.1016/j.jscai.2022.100432

**Published:** 2022-08-13

**Authors:** Raúl Moreno, David E. Kandzari, Ajay J. Kirtane, Stephan Windecker, Azeem Latib, Elvin Kedhi, Roxana Mehran, Matthew J. Price, Daniel I. Simon, Stephen G. Worthley, Douglas Spriggs, Thaddeus Tolleson, Tamim Nazif, Harsh Golwala, Nathan H. Kander, Houng B. Liew, Gennaro Sardella, Corrado Tamburino, Te-Hsin Lung, Cecile Mahoney, Gregg W. Stone

**Affiliations:** a Hospital La Paz, Madrid, Spain; b Piedmont Heart Institute, Atlanta, Georgia; c Columbia University Irving Medical Center/NewYork-Presbyterian Hospital, New York, New York; d Cardiovascular Research Foundation, New York, New York; e University Hospital, University of Bern, Bern, Switzerland; f Montefiore Medical Center, New York, New York; g Free University of Brussels, Brussels, Belgium; h Silesian Medical University, Katowice, Poland; i The Zena and Michael A. Weiner Cardiovascular Institute, Icahn School of Medicine at Mount Sinai, New York, New York; j Scripps Clinic, La Jolla, California; k University Hospitals Cleveland Medical Center, Cleveland, Ohio; l GenesisCare, Alexandria, Australia; m Morton Plant Hospital, Clearwater, Florida; n University of Texas Health East Texas, Tyler, Texas; o Oregon Health and Science University Hospital, Portland, Oregon; p OhioHealth Riverside Methodist Hospital, Columbus, Ohio; q Queen Elizabeth II Hospital, Grande Prairie, Alberta, Canada; r Umberto I – Policlinico di Roma, Rome, Italy; s Presidio Ospedaliero Ferrarotto Alessi, Catania, Italy; t Medtronic, Santa Rosa, California

**Keywords:** bleeding risk, coronary stenting, percutaneous coronary intervention

## Abstract

**Background:**

Small reference vessel diameters (RVDs) are a predictor of ischemic events after coronary stenting. Among patients at high bleeding risk (HBR) precluding long-term dual antiplatelet therapy (DAPT), those with small vessel disease (SVD) constitute an especially high-risk subgroup. Here, we evaluated the results of a durable-polymer, coronary zotarolimus-eluting stent (ZES) for the treatment of patients with SVD at HBR with 1-month DAPT.

**Methods:**

In the prospective, multicenter Onyx ONE (One-Month DAPT) Clear study, 1506 patients at HBR treated with a ZES that discontinued DAPT at 30 days were included. The clinical outcomes of patients undergoing treatment of lesions with an RVD of ≤2.5 mm (SVD group, as determined by the angiographic core laboratory) were compared with patients without SVD. The primary end point was the composite of cardiac death or myocardial infarction between 1 and 12 months.

**Results:**

Small vessel diameter treatment was performed in 489 (32.5%) patients. Patients with SVD were more likely to be women, have undergone a previous percutaneous intervention, and have multivessel coronary artery disease than patients without SVD. There were no significant differences in lesion, device, or procedural success between the groups. The Kaplan-Meier rate estimate of the primary end point was 8.5% and 6.8% in patients with SVD and those without SVD, respectively (*P* = .425). No significant differences were found in any secondary end point. The Kaplan-Meier rate of stent thrombosis was 0.6% and 0.8% in patients with SVD and those without SVD, respectively (*P* = .50).

**Conclusions:**

Among patients at HBR treated with a ZES and 1-month DAPT, those with SVD had favorable 12-month ischemic and bleeding outcomes, which were comparable with those of patients with larger caliber vessels.

## Highlights

• Small vessels are a major risk factor for ischemic events after PCI.
• Balancing ischemic versus bleeding risks in HBR patients remains challenging.
• One-month DAPT after PCI is an emerging strategy in HBR.
• HBR patients with small vessels received the Onyx drug-eluting stent.

## Central Illustration

**Figure:**
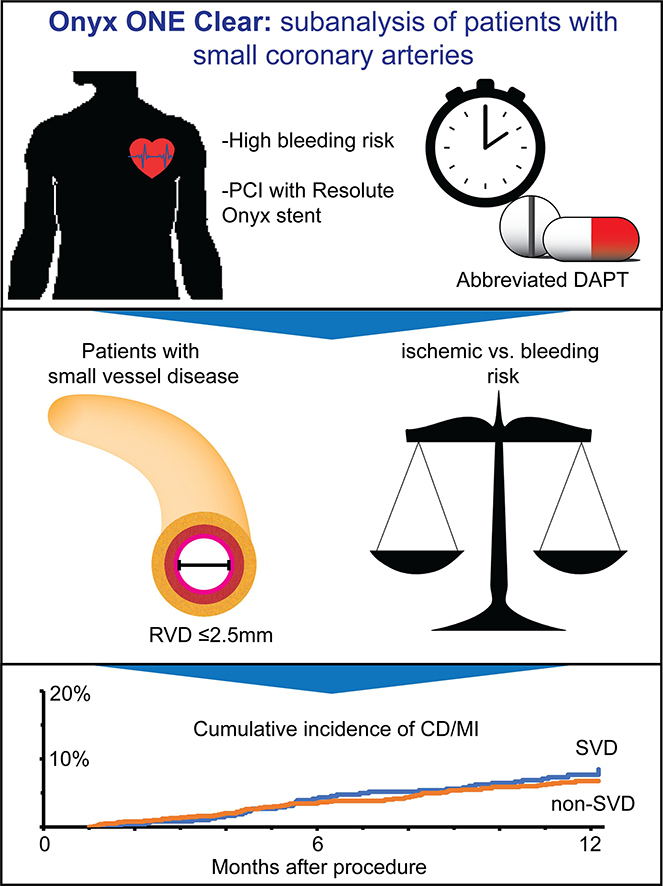

## Introduction

Vessel diameter is a well-recognized risk factor for ischemic events after percutaneous coronary intervention (PCI), with small vessels having increased rates of target vessel revascularization (TVR) procedures and stent thrombosis (ST).^1^, ^2^, ^3^ Thus, patients with small coronary arteries and increased risk of bleeding, requiring a short dual antiplatelet therapy (DAPT) duration, comprise a particularly high-risk group.

Onyx ONE (One-Month DAPT) Clear was a large-scale, prospective, multicenter, nonrandomized study that evaluated the safety and effectiveness of 1-month DAPT followed by single antiplatelet therapy (SAPT) in patients at high bleeding risk (HBR) undergoing PCI with Resolute Onyx drug-eluting stents.^4^ No patient was excluded based on angiographic criteria or clinical presentation. Thus, many patients undergoing PCI of small vessels were included.

In the present analysis based on Onyx ONE Clear, the clinical outcomes of patients undergoing PCI for small vessel disease (SVD) were compared with those of patients undergoing PCI of only larger vessel diameters.

## Methods

### Study patients

Briefly, Onyx ONE Clear was a prospective, multicenter, nonrandomized study designed to evaluate the clinical safety and effectiveness of 1-month DAPT in patients at HBR after implantation of Resolute Onyx zotarolimus-eluting stents (ZES; Medtronic Cardiovascular, Plc). Patients were entered into Onyx ONE Clear from 2 sequential investigations with similar enrollment criteria: the Onyx ONE randomized controlled trial (RCT) and the Onyx ONE United States or Japan study.^5^ The Onyx ONE RCT included 1018 patients and the Onyx ONE United States or Japan study included 752 patients who received at least 1 Resolute Onyx stent. These 2 studies included “1-month clear” patients who were free of any events in the first month after stenting that could preclude early cessation of DAPT. These events included nonperiprocedural myocardial infarction (MI), repeat coronary revascularization, stroke, definite or probable ST, and death. Onyx ONE Clear patients were required to adhere to DAPT for the entire 1-month period, defined as no interruption of aspirin or P2Y_12_ inhibitor for >3 cumulative days during the first month after the index procedure. For the 1-month clear analysis, the criteria were extended to 1 month beyond a planned staged procedure when performed. A total of 1506 patients fulfilled these criteria and then discontinued DAPT per protocol; these patients comprised the study population of Onyx ONE Clear. An independent core laboratory evaluated all angiograms at baseline and at the time of ischemic events.

The results and clinical outcomes of patients undergoing PCI of ≥1 lesions with a reference vessel diameter (RVD) of ≤2.5 mm (SVD group) and those without any such lesions (ie, all lesions with an RVD of >2.5 mm, non-SVD group) were compared. Preprocedural RVDs were determined using a quantitative coronary analysis by the angiographic core laboratory and were used to determine subgroup classification (SVD vs non-SVD).

### Study end points

The end points of the present analysis were similar to those of the main study.^4^ The primary end point was the composite of cardiac death (CD) or MI between 1 and 12 months among patients in the 1-month clear population. The secondary end points included target lesion failure (TLF; characterized by CD, target vessel MI, or target lesion revascularization [TLR]), target vessel failure (characterized by CD, target vessel MI, or clinically driven repeat TVR), all-cause death, CD, MI, TLR, TVR, definite or probable ST, stroke, and bleeding.

MI was defined according to the Third Universal Definition of Myocardial Infarction.^6^ ST and bleeding events were defined according to Academic Research Consortium and Bleeding Academic Research Consortium criteria, respectively.^7^^,^^8^ Acute device, lesion, and procedural success were also assessed. Lesion success was defined as an achievement of <30% residual stenosis and Thrombolysis in Myocardial Infarction (TIMI) 3 flow. Device success was defined as lesion success with the assigned study device. Procedural success was defined as lesion success and the absence of in-hospital major adverse cardiac events (MACE). An independent clinical events committee adjudicated all the primary and secondary end points.

### Statistical analysis

All analyses of clinical outcomes were performed at the patient level. Note that numerous patients had >1 lesion treated, some of whom had lesion(s) with an RVD of ≤2.5 mm and some had lesion(s) with an RVD of >2.5 mm. If a patient had at least 1 RVD of ≤2.5 mm, they were categorized in the SVD group in this analysis. Conversely, if a patient only had lesion(s) with an RVD of >2.5 mm, they were categorized in the non-SVD group. Categorical data are reported as percentages (counts) and were compared between the groups using the Fisher exact test. Continuous data are reported as means ± standard deviations and were compared between the groups using the 2-sample *t* test. Cumulative incidence curves with Kaplan-Meier rate estimates were generated for the primary end point (CD or MI), TLR, and ST. For the comparison of clinical outcomes, *P* values were calculated using Cox regression, in which an outcome was regressed on the SVD status and propensity score based on age, sex, previous PCI, diabetes, multivessel coronary artery disease, acute coronary syndrome, and maximum lesion length. All statistical analyses were performed using SAS (version 9.4; SAS Institute).

## Results

### Comparison of patients with and without SVD

Among the 1506 patients included in Onyx ONE Clear, 16 (1.0%) did not have their RVD analyzed before the procedure and were, therefore, excluded from subsequent analyses. Among the remaining 1490 patients, 489 (32.8%) underwent PCI of ≥1 small vessel, comprising the SVD group. In this group, a total of 748 lesions were treated, including 534 with an RVD of ≤2.5 mm and 176 with an RVD of >2.5 mm. In the non-SVD group, 1001 (67.2%) patients underwent PCI of 1201 lesions, all of which had an RVD of >2.5 mm. The patients with SVD had a mean RVD of 2.44 ± 0.34 mm, whereas the patients without SVD had a mean RVD of 3.05 ± 0.40 mm (*P* < .001).

Table 1 shows the comparison of the baseline characteristics of the patients with SVD and those without SVD. The patients with SVD were more likely to be women and had a higher prevalence of previous PCI, insulin-dependent diabetes mellitus, multivessel disease, and non-ST-elevation MI as the clinical indication for the index procedure. The HBR criteria were similar between the patients with SVD and those without SVD (Supplemental Table S1).

**Table 1:** Baseline demographics and clinical characteristics.

	SVD RVD ≤2.5 mm (N = 489)	No SVD RVD >2.5 mm (N = 1001)	*P* value
Age, y	74.7 ± 9.1	73.6 ± 9.7	.048
Female	38.7% (189/489)	29.2% (292/1001)	<.001
BMI, kg/m^2^	28.1 ± 5.6	28.2 ± 5.8	.66
Previous MI	27.4% (134/489)	25.6% (256/1001)	.45
Previous PCI	36.4% (178/489)	26.9% (269/1001)	<.001
Previous CABG	13.1% (64/489)	12.6% (126/1001)	.80
Hyperlipidemia	74.8% (366/489)	70.9% (710/1001)	.12
Hypertension	84.0% (411/489)	84.0% (841/1001)	> .999
Stroke or TIA	14.1% (69/489)	14.2% (142/1001)	>.999
Cardiac admissions within 30 d prior to index procedure	10.2% (50/489)	9.6% (96/1001)	.71
COPD	14.1% (69/489)	11.9% (119/1001)	.25
Peripheral vascular disease	10.8% (53/489)	10.4% (104/1001)	.79
Atrial fibrillation	36.0% (176/489)	35.3% (353/1001)	.82
OAC use at discharge	35.4% (173/489)	35.7% (357/1001)	.954
Current smoker	9.0% (44/488)	9.8% (97/994)	.71
Diabetes mellitus	42.5% (208/489)	37.9% (379/1001)	.09
Type I	1.2% (6/489)	0.4% (4/1001)	.09
Type II	41.3% (202/489)	37.5% (375/1001)	.16
Insulin dependent	17.6% (86/489)	11.6% (116/1001)	.002
Serum creatinine, μmol/L	123.1 ± 172.5	123.7 ± 137.0	.95
Left ventricular ejection fraction, %	53.0 ± 12.2	52.4 ± 12.5	.47
Left ventricular ejection fraction ≤ 35%	11.8% (43/364)	13.5% (101/748)	.45
Multivessel disease	55.6% (272/489)	46.9% (469/1001)	.002
Clinical evidence (prompted index procedure)	96.1% (470/489)	95.4% (955/1001)	.59
Silent ischemia	11.1% (52/470)	10.8% (103/955)	.86
Stable angina	37.2% (175/470)	42.6% (407/955)	.06
Unstable angina	23.6% (111/470)	21.8% (208/955)	.46
Myocardial infarction	28.1% (132/470)	24.8% (237/955)	.20
STEMI	3.2% (15/470)	4.7% (45/955)	.21
Non-STEMI	24.9% (117/470)	20.1% (192/955)	.04
Acute coronary syndrome	51.7% (243/470)	46.6% (445/955)	.07
Positive functional study	3.9% (19/489)	4.6% (46/1001)	.59

Values are % (n/N) or mean ± SD.

BMI, body mass index; CABG, coronary artery bypass grafting; COPD, chronic obstructive pulmonary disease; MI, myocardial infarction; OAC, oral anticoagulant; PCI, percutaneous coronary intervention; RVD, reference vessel diameter; STEMI, ST-segment-elevation myocardial infarction; SVD, small vessel disease; TIA, transient ischemic attack.

**Figure 1:**
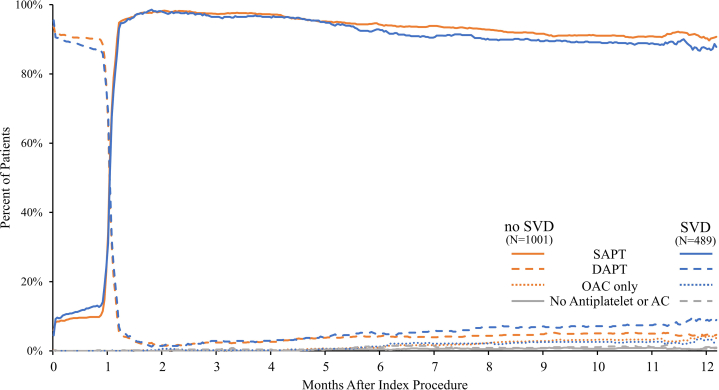
**The use of dual antiplatelet therapy, single antiplatelet therapy, and oral anticoagulants in Onyx ONE(One-Month DAPT)Clear patients with and without small vesseldiseasethrough 12 months.** SAPT, DAPT, and OAC use are illustrated comparing patients with and without SVD. AC, anticoagulant; DAPT, dual antiplatelet therapy; OAC, oral anticoagulant; SAPT, single antiplatelet therapy; SVD, small vessel disease.

The use of DAPT, SAPT, and oral anticoagulation are illustrated in Figure 1. The discontinuation of DAPT after 1 month was similar between the patient groups, and by 2 months, more than 97.0% of the patients in both the groups had transitioned to SAPT. By 12 months after PCI, 87.7% and 90.0% of the patients with SVD and those without SVD, respectively, were adherent to SAPT (*P* = .197) (Supplemental Table S2).

### Procedural results

As shown in Table 2, all the patients received only Resolute Onyx ZES. The number of lesions and vessels treated, number of stents implanted, and total stent length were higher in the patients with SVD than in those without SVD. The prevalence of moderate or severe calcification as well as the American College of Cardiology/American Heart Association lesion class B2/C were higher in the patients without SVD than in those with SVD. The amount of contrast agent used and the number of staged procedures were higher in the patients with SVD than in those without SVD. In contrast, image guidance using intravascular ultrasound or optical coherence tomography was lower in the patients with SVD than in those without SVD. Quantitative coronary angiography (Supplemental Table S3) showed that the baseline mean lesion lengths were shorter, and as expected, the RVD and minimum lumen diameters were smaller in the patients with SVD than in those without SVD. However, the percent diameter stenosis (DS) was similar in both the groups. After the procedure, the minimum lumen diameters and acute gain were significantly greater in the non-SVD group (Supplemental Table S3). The postprocedural in-stent DS was greater in the patients without SVD; however, there was no significant difference in in-segment DS between the groups. Furthermore there were no significant differences in lesion, device, or procedural success between the groups (Table 2), although there was a trend toward higher lesion and device success rates in the patients with SVD.

**Table 2:** Procedural characteristics.

	SVD RVD ≤ 2.5 mm (N = 489 patients) (N = 748 lesions)	No SVD RVD > 2.5 mm (N = 1001 patients) (N = 1201 lesions)	*P* value
Location of vascular access			.08
Femoral	37.4% (194/519)	31.8% (324/1020)	
Radial	61.8% (321/519)	67.6% (690/1020)	
Brachial	0.8% (4/519)	0.6% (6/1020)	
Vessel location (per patient)			
LAD	59.1% (289/489)	49.7% (497/1001)	<.001
LCX	38.4% (188/489)	22.7% (227/1001)	<.001
RCA	31.1% (152/489)	35.8% (358/1001)	.081
Left main	1.0% (5/489)	1.2% (12/1001)	> .999
Arterial or venous graft	2.2% (11/489)	4.9% (49/1001)	.016
Total procedure time, min	41.9 ± 28.5	41.8 ± 30.5	.94
Total fluoroscopy time, min	16.2 ± 11.9	15.3 ± 12.5	.22
Staged procedure	6.1% (30/489)	1.9% (19/1001)	<.001
Hospital length of stay, d	1.9 ± 3.5	1.8 ± 3.7	.72
Total contrast volume, mL	173.2 ± 87.7	157.2 ± 69.3	<.001
Image-guided IVUS or OCT	13.7% (67/489)	20.1% (201/1001)	.003
Number of vessels treated per patient	1.3 ± 0.5	1.1 ± 0.4	<.001
Stent diameter per patient, mm	2.6 ± 0.3	3.2 ± 0.4	<.001
Moderate or severe calcification	43.2% (320/740)	54.1% (649/1199)	<.001
B2/C lesion class	75.1% (562/748)	81.3% (977/1201)	.001
Total stent length, mm			
Per patient	41.5 ± 30.5	34.8 ± 23.7	<.001
Per lesion	25.4 ± 14.7	26.0 ± 13.8	.33
Number of lesions treated per patient	1.5 ± 0.7	1.2 ± 0.5	<.001
Number of lesions with an RVD ≤ 2.5 mm treated per patient	1.2 ± 0.4	0	N/A
Number of stents implanted			
Per patient	2.0 ± 1.2	1.5 ± 0.8	<.001
Per lesion	1.2 ± 0.5	1.2 ± 0.4	.09
Lesion success	95.9%	93.8%	.060
Device success	94.5%	92.5%	.091
Procedural success	88.2%	88.7%	.793

Values are %, % (n/N), or mean ± SD.

IVUS, intravascular ultrasound; LAD, left anterior descending; LCX, left circumflex; N/A, not applicable; OCT, optical coherence tomography; RCA, right coronary artery; RVD, reference vessel diameter; SVD, small vessel disease.

### Clinical outcomes

**Figure 2:**
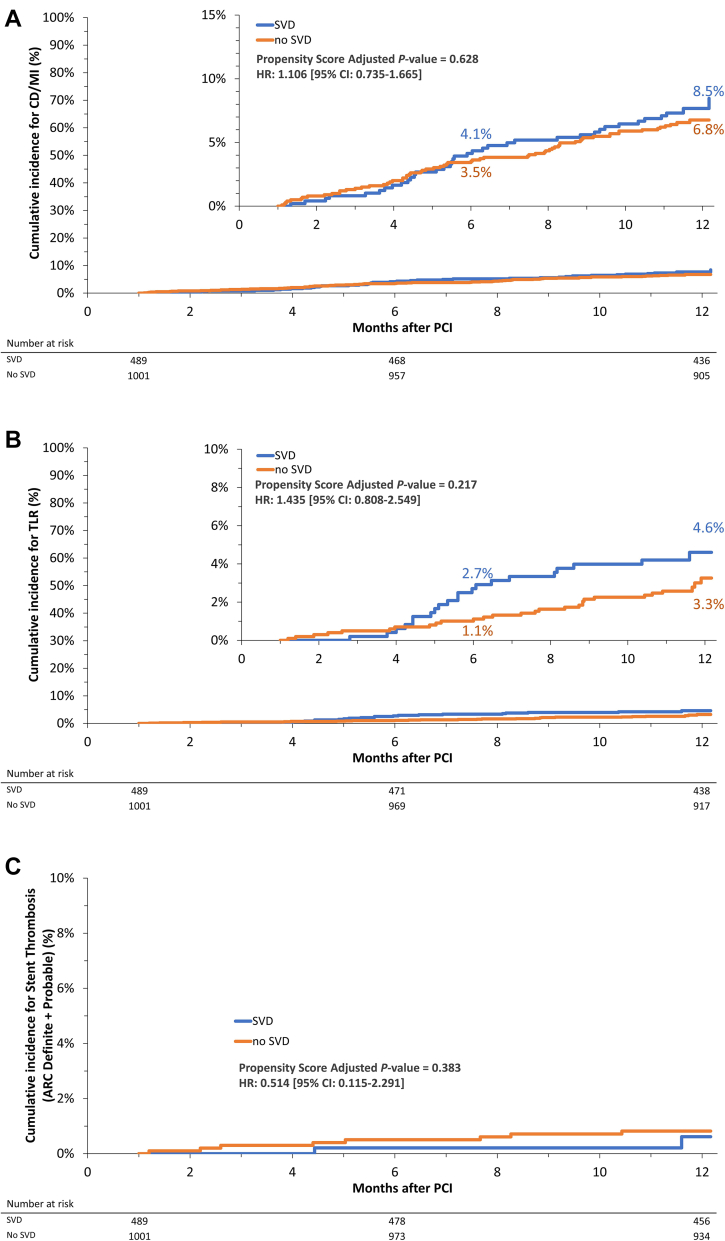
**Kaplan-Meier curves (inset, zoom in) for time to first events from 1 to 12 months for patients with and without small vessel** disease**.** (**A**) Cardiac death and myocardial infarction, (**B**) target lesion revascularization, and (**C**) probable and definite stent thrombosis. ARC, Academic Research Consortium; CD, cardiac death; MI, myocardial infarction; PCI, percutaneous coronary intervention; SVD, small vessel disease; TLR, target lesion revascularization.

The Kaplan-Meier curve estimates of clinical outcomes at 12 months are shown in Table 3. The Kaplan-Meier estimate of the primary end point (composite of CD or MI) between 1 and 12 months was comparable between the SVD and non-SVD groups (8.5% vs 6.8%, respectively; hazard ratio, 1.106; 95% CI, 0.735-1.665; propensity score-adjusted Cox regression *P* = .63) (Figure 2A).

**Table 3:** Kaplan-Meier rate estimates between 1 and 12 months in patients with versus without small vessel disease.

	SVD RVD ≤2.5 mm (N = 489 patients)	No SVD RVD >2.5 mm (N = 1001 patients)	*P* value using Cox regression	Propensity score-adjusted *P* value using Cox regression
Primary end point: CD/MI	8.5% (37)	6.8% (65)	.425	.628
Secondary end point: TLF	10.1% (45)	7.9% (74)	. 210	.325
Target vessel failure	12.0% (52)	8.3% (77)	.054	.100
All-cause death	6.3% (29)	6.2% (59)	.934	.925
CD	3.1% (14)	2.6% (25)	.645	.621
Non-CD	3.3% (15)	3.7% (34)	.798	.608
MI (third UDMI)	6.3% (27)	4.5% (43)	.283	.494
Clinically driven TLR	4.6% (21)	3.3% (28)	.117	.217
Clinically driven TVR	6.6% (28)	4.0% (35)	.042	.085
All revascularizations	8.2% (36)	5.1% (46)	.025	.068
Definite or probable late ST	0.6% (2)	0.8% (8)	.500	.383
Stroke	1.3% (6)	1.6% (16)	.649	.513
Bleeding				
All BARC	15.3% (71)	13.0% (123)	.192	.338
BARC 2-5	14.7% (68)	11.3% (106)	.053	.109
BARC 3-5	6.0% (26)	3.5% (34)	.077	.150

Values are % (n).

BARC, Bleeding Academic Research Consortium; CD, cardiac death; MI, myocardial infarction; RVD, reference vessel diameter; ST, stent thrombosis; SVD, small vessel disease; TLF, target lesion failure; TLR, target lesion revascularization; TVR, target vessel revascularization; UDMI, universal definition of myocardial infarction.

**Central Illustration:**
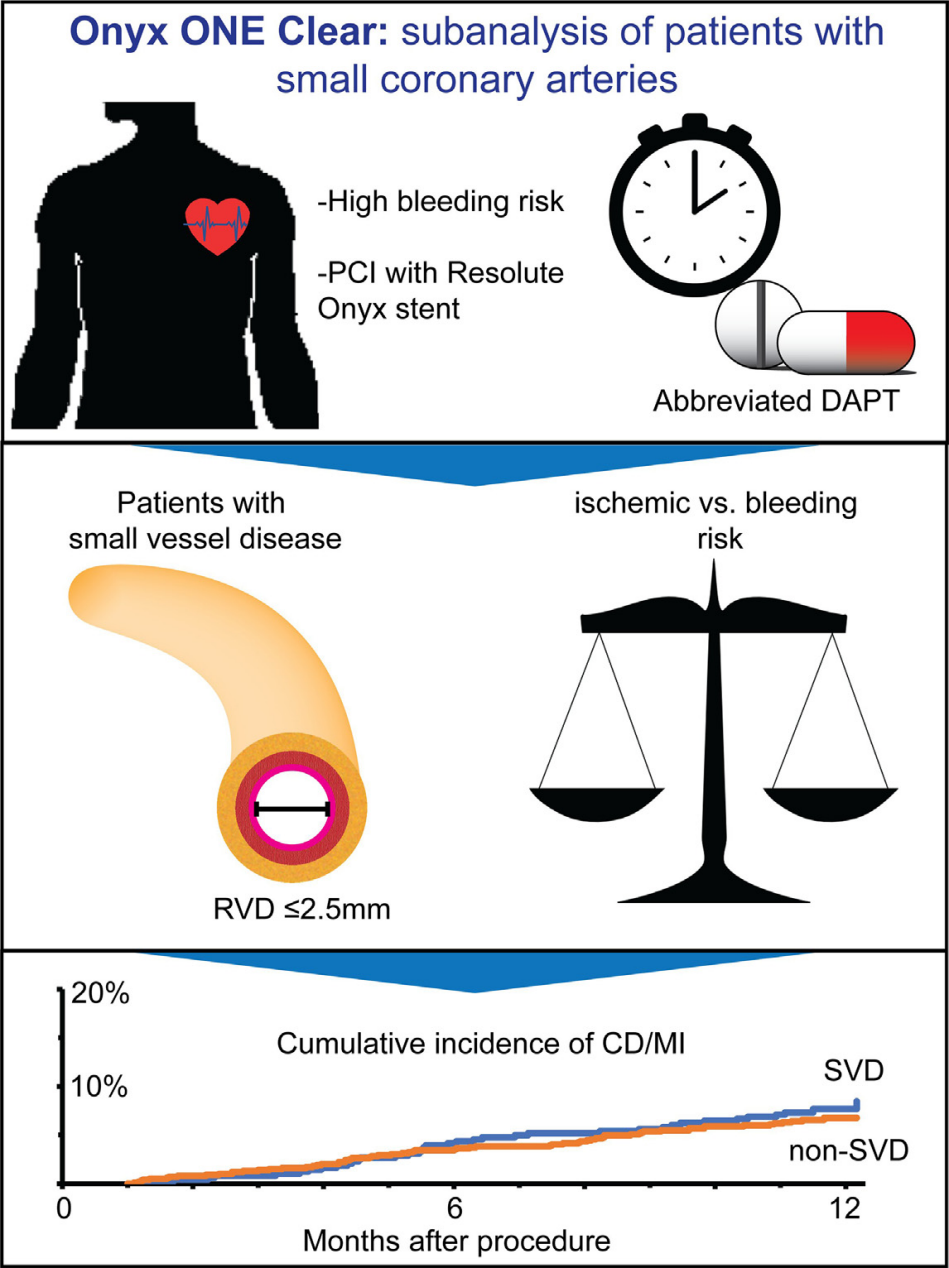
Onyx ONE (One-Month DAPT) Clear: a subanalysis of patients with small coronary arteries. CD, cardiac death; DAPT, dual antiplatelet therapy; MI, myocardial infarction; PCI, percutaneous coronary intervention; RVD, reference vessel diameter; SVD, small vessel disease; DAPT, dual antiplatelet therapy.

No significant differences were found in the rates of TLF, target vessel failure, or their individual components, including MI, between the patients with SVD and those without SVD (Table 3). Although the rate of repeat revascularization was higher in the patients with SVD than in those without SVD, after adjusting for propensity score, this difference was not significant. The differences in the rates of clinically driven TLR (Figure 2B) and ST (Figure 2C) between the patients with SVD and those without SVD were not significant (Table 3). The Kaplan-Meier curves for TLR and ST are shown in Figure 2. The Kaplan-Meier rate estimates of bleeding significantly were also not different between the patients with SVD and those without SVD (Table 3).

## Discussion

The main findings of the present study are as follows: (1) among patients at HBR treated with Resolute Onyx stent implantation who were included in the Onyx ONE Clear study, the treatment of SVD was frequent (approximately one-third of the patients); (2) the device success rates were similar in the patients with SVD and those without SVD; (3) the composite rates of CD or MI between 1 and 12 months, in addition to the secondary end points, were similar in the patients with SVD and those without SVD; and (4) among the patients with SVD, the rate of ST between 1 and 12 months was low despite 1-month DAPT. We note that these results were obtained based on PCI using only the Resolute Onyx stent and may not apply to other stent platforms.

The recommended antithrombotic therapy after coronary stenting typically includes DAPT for at least 6 months in patients with chronic stable coronary artery disease^9^ and 12 months in those with acute coronary syndromes.^10^ However, reduction in DAPT duration to 3 months or even 1 month is frequently needed in clinical practice, especially when the risk of bleeding is high.^9^^,^^10^ The Resolute Onyx coronary stent was shown to be safe in patients at HBR with 1-month DAPT in both the Onyx ONE RCT and Onyx ONE Clear studies.^4^^,^^5^ Furthermore, the Resolute Onyx ZES is highly biocompatible, with thin struts (81 μm) that are circumferentially covered with a 5.6-μm layer of the biocompatible, durable-polymer BioLinx, which promotes endothelialization and vascular healing.^11^^,^^12^ In the BIONYX (Bioresobable Polymer ORSIRO Versus Durable Polymer RESOLUTE ONYX Stents) randomized trial, the Resolute Onyx stent had a significantly lower rate of ST at 12 months than a biodegradable polymer-based sirolimus-eluting stent.^13^ Similarly, in the Onyx ONE Clear study, which included only patients at HBR using a 1-month DAPT regimen, the risk of events from 1 to 12 months was low, with rates of TLF and ST of 8.1% and 0.7%, respectively.^4^

Small vessel disease have been associated with a high rate of events, especially repeat revascularization procedures and ST.^1^, ^2^, ^3^ Because late lumen loss is independent of vessel size,^13^ binary in-stent restenosis is more frequent in patients with small RVDs.^14^ This translates to a higher incidence of adverse ischemic end points, not only repeat revascularization procedures but also MI and ST after PCI of lesions with small RVDs (Central Illustration).^14^, ^15^, ^16^, ^17^, ^18^

In the present analysis based on Onyx ONE Clear, the patients with SVD, characterized by an RVD of ≤2.5 mm, had favorable clinical outcomes. Repeat revascularization procedures were more frequent in the patients with SVD, but not of the target lesion. The patients with SVD had a higher prevalence of multivessel disease, received a greater number of coronary stents, and had longer total stent lengths to treat more lesions and more vessels. The patients with SVD were more frequently diabetics receiving insulin and had a higher prevalence of previous PCI, which can also increase the risk of new revascularizations. Notably, the risk of ST between 1 and 12 months was low in the patients with SVD, even numerically lower than that in the patients without SVD (0.6% vs 0.8%, respectively). The risk of MI was comparable between the patients with SVD and those without SVD (6.3% vs 4.5%, respectively), underscoring the relative safety of the Resolute Onyx stent in patients with an RVD of ≤2.5 mm.

These results demonstrate that in patients at HBR undergoing PCI for SVD and for whom short-term DAPT therapy may improve the risk-benefit balance, the Resolute Onyx stent offers favorable clinical results, comparable with those in patients with larger vessels, without increasing the risk of clinical events. This is notable because patients with SVD are, in general, at higher risk of ischemic complications. A study investigating the safety and efficacy of the Resolute Onyx 2.0-mm ZES used in the treatment of coronary stenoses in vessels with a diameter of <2.25 mm reported 5.0%, 2.0%, and 3.0% rates of TLF, target lesion revascularization, and target vessel-related MI at 12 months, respectively, with no cases of ST.^19^ However, it should be noted that the overall lesion complexity was higher in the Onyx ONE Clear study.^1^

## Conclusions

A high proportion of patients at HBR treated with Resolute Onyx ZES followed by 1-month DAPT in the Onyx ONE Clear trial had SVD. Among these patients, the clinical outcomes between 1 and 12 months were favorable and comparable with outcomes among patients with larger vessels, supporting a short DAPT approach after small-vessel PCI using this stent.
