# Supplementary material for: Coronary Stenting in High Bleeding Risk Patients With Small Coronary Arteries Followed by One-Month Dual Antiplatelet Therapy: Onyx ONE Clear
Source: J Soc Cardiovasc Angiogr Interv. 2022 Aug 13;1(6):100432. doi: 10.1016/j.jscai.2022.100432 (PMC11308796; doi:10.1016/j.jscai.2022.100432)
Supplement: Supplementary Data [file mmc1.docx]

**Supplemental Table S1.**High bleeding risk criteria

|  | | | |
| --- | --- | --- | --- |
|  | **SVD**  **RVD ≤ 2.5mm (N=489)** | **no SVD**  **RVD > 2.5mm (N=1001)** | **P-value** |
| Mean number of HBR criteria | 1.6 ± 0.9 | 1.6 ± 0.7 | 0.51 |
| Oral anticoagulation to continue after PCI | 40.3% (197) | 41.5% (415) | 0.70 |
| Age ≥ 75 years | 61.1% (299) | 58.0% (581) | 0.26 |
| Hgb <11 g/dl (or transfusion within 4 weeks before procedure) | 14.7% (72) | 14.1% (141) | 0.75 |
| Prior intracerebral bleed | 2.0% (10) | 1.6% (16) | 0.53 |
| Stroke in previous 12 months | 1.8% (9) | 3.0% (30) | 0.23 |
| Hospital admission for major bleeding in prior 12 months | 3.7% (18) | 2.4% (24) | 0.18 |
| Non skin cancer diagnosed or treated within 3 years | 7.8% (38) | 7.3% (73) | 0.75 |
| NSAID (other than aspirin) or steroids for ≥ 30 days after PCI | 3.5% (17) | 3.0% (30) | 0.64 |
| Planned surgery in next 12 months requiring interruption of DAPT | 6.1% (30) | 6.9% (69) | 0.66 |
| Creatinine clearance <40 ml/min | 12.3% (60) | 12.5% (125) | 0.93 |
| Thrombocytopenia (platelets <100,000/mm^3^) | 1.8% (9) | 1.6% (16) | 0.83 |
| Severe chronic liver disease | 0.2% (1) | 1.3% (13) | 0.045 |
| Expected non-compliance to prolonged DAPT | 4.7% (23) | 4.0% (40) | 0.58 |
| HBR: high bleeding risk; RVD: reference vessel diameter; PCI: percutaneous coronary intervention; Hgb: hemoglobin; DAPT: dual antiplatelet therapy; NSAID: nonsteroidal anti-inflammatory drug | | | |

**Supplemental Table S2.**

**Prescribed medications at two and 12 months**

|  | | | |
| --- | --- | --- | --- |
| **Antiplatelet/Anticoagulant** | **SVD**  **RVD ≤ 2.5mm**  **(N=489 Patients)** | **no SVD**  **RVD > 2.5mm**  **(N=1001 Patients)** | **P-value** |
| **At 2 months** | | | |
| DAPT ± OAC | 3.1% | 2.9% | 0.871 |
| SAPT | 96.7% | 97.0% | 0.752 |
| SAPT (no OAC) | 59.4% | 60.1% | 0.822 |
| Aspirin | 40.0% | 41.6% | 0.575 |
| P2Y12 inhibitor | 19.5% | 18.4% | 0.621 |
| SAPT + OAC | 37.3% | 36.9% | 0.909 |
| OAC + aspirin | 16.2% | 14.1% | 0.312 |
| OAC + P2Y12 inhibitor | 21.1% | 22.8% | 0.466 |
| OAC | 38.5% | 37.3% | 0.690 |
| Vitamin K antagonist | 8.6% | 10.5% | 0.268 |
| Other OAC | 29.9% | 26.9% | 0.242 |
| **At 12 months** | | | |
| DAPT ± OAC | 8.4% | 5.6% | 0.064 |
| SAPT | 87.7% | 90.0% | 0.197 |
| SAPT (no OAC) | 53.1% | 55.6% | 0.389 |
| Aspirin | 37.0% | 39.2% | 0.445 |
| P2Y12 inhibitor | 16.1% | 16.4% | 0.938 |
| SAPT + OAC | 34.6% | 34.4% | 0.952 |
| OAC + aspirin | 14.5% | 14.8% | 0.936 |
| OAC+ PYP12 inhibitor | 20.0% | 19.6% | 0.886 |
| OAC | 37.9% | 38.8% | 0.769 |
| Vitamin K antagonist | 8.4% | 10.2% | 0.288 |
| Other OAC | 29.5 | 28.6% | 0.753 |

**Supplemental Table S3.** Pre- and Post-Procedural Lesion Characteristics

| **Lesion Characteristics** | **SVD**  **RVD ≤ 2.5mm**  **(N=489 Patients)**  **(N=748 Lesions)** | **no SVD**  **RVD > 2.5mm**  **(N=1001 Patients)**  **(N=1201 Lesions)** | **P-value** |
| --- | --- | --- | --- |
| **Pre-Procedural** |  |  |  |
| Lesion length (mm) | 19.98 ± 12.44 | 21.27 ± 13.35 | 0.033 |
| Reference vessel diameter (mm) | 2.44 ± 0.34 | 3.05 ± 0.40 | <0.001 |
| Minimum lumen diameter (mm) | 0.79 ± 0.33 | 0.95 ± 0.43 | <0.001 |
| Diameter stenosis (%) | 67.59 ± 12.83 | 68.76 ± 13.53 | 0.060 |
|  |  |  |  |
| **Post-procedural** |  |  |  |
| Minimum lumen diameter (mm) |  |  |  |
| In-stent | 2.28 ± 0.34 | 2.77 ± 0.43 | <0.001 |
| In-segment | 2.02 0.38 | 2.49 ± 0.44 | <0.001 |
| Proximal edge | 2.52 ± 0.47 | 3.16 ± 0.55 | <0.001 |
| Distal edge | 2.10 ± 0.38 | 2.58 ± 0.49 | <0.001 |
| Diameter stenosis (%) |  |  |  |
| In-stent | 8.89 ± 9.23 | 10.78 ± 8.70 | <0.001 |
| In-segment | 19.52 ± 10.12 | 20.04 ± 9.14 | 0.255 |
| Acute Gain (mm) |  |  |  |
| In-stent | 1.49 ± 0.40 | 1.82 ± 0.51 | <0.001 |
| In-segment | 1.23 ± 0.41 | 1.53 ± 0.52 | <0.001 |

|  |
| --- |
